# Protection from the Embryopathic Effects of 7-Hydroxymethyl-12-Methylbenz(a)Anthracene by 2-Methyl-1,2-bis-(3-Pyridyl)-1-Propanone (Metopirone,CIBA) and β-Diethylaminoethyldiphenyl-n-Propyl Acetate (SKF 525-A)

**DOI:** 10.1038/bjc.1970.66

**Published:** 1970-09

**Authors:** C. C. Bird, Allison M. Crawford, A. R. Currie, B. Fiona Stirling

## Abstract

**Images:**


### PROTECTION FROM THE EMBRYOPATHIC EFFECTS OF 7-HYDROXYMETHYL-12-METHYLBENZ(*a*)ANTHRACENE BY 2-METHYL-1,2-BIS-(3-PYRIDYL)-1-PROPANONE (METOPIRONE, CIBA) AND β-DIETHYLAMINOETHYLDIPHENYL-*n*-PROPYL ACETATE (SKF 525-A)

C. C. BIRD, ALLISON M. CRAWFORD, A. R. CURRIE AND B. FIONA STIRLING

*From the Department of Pathology, University of Aberdeen*

Received for publication May 15, 1970

**SUMMARY.—Pretreatment with Metopirone (40 mg.) or SKF 525-A (2 mg./100 g. maternal body weight) protected against the embryotoxic and teratogenic actions of 7-OHM-12-MBA (2·5 mg/100 g. maternal body weight) in the Sprague-Dawley rat. At the doses administered SKF-525-A was a more efficient protector than Metopirone. The adrenocorticolytic actions of 7-OHM-12-MBA in the maternal adrenal glands were also prevented by these compounds and a close correlation existed between the degree of protection of the maternal adrenals and of the foetuses. It is suggested that the ultimate embryopathic substance is a metabolite of 7-OHM-12-MBA.**

7,12-Dimethylbenz(*a*)anthracene (DMBA) and its metabolite 7-hydroxymethyl-12-methylbenz(*a*)anthracene (7-OHM-12-MBA) are powerful adrenocorticolytic agents in the Sprague-Dawley rat (Boyland, Sims and Huggins, 1965; Wheatley *et al.*, 1966). These effects can be prevented by pretreatment with 2-methyl-1,2-bis-(3-pyridyl)-1-propanone (Metopirone, CIBA) (Currie, Helfenstein and Young, 1962; Dao and Tanaka, 1963; Wong and Warner, 1964; Wheatley, 1968*a*), or with *β*-diethylaminoethyldiphenyl-*n*-propyl acetate (SKF 525-A) (Wheatley, 1968*b*), both of which affect the metabolism of DMBA and 7-OHM-12-MBA by the liver; they also inhibit corticosteroid synthesis (Chart *et al.*, 1958; Huffman and Azarnoff, 1967).

Recently we described the embryopathic effects of DMBA and 7-OHM-12-MBA in the Sprague-Dawley rat (Currie *et al.*, 1970). Treatment with a single intravenous dose of 7-OHM-12-MBA (2·5 mg./100 g. maternal body weight) at day 8 of pregnancy produced 100% resorptions; the same dose at day 13 caused characteristic axial skeleton malformations in 100% of surviving foetuses and there was no increase in the resorption rate. Here we report the results of experiments which show that Metopirone and SKF 525-A prevent the embryopathic effects of 7-OHM-12-MBA.

### MATERIALS AND METHODS

Non-parous female Sprague-Dawley rats, 200–300 g. in weight, were obtained from Oxford Laboratory Animal Colonies, Bicester, Oxon. The male Sprague-Dawley rats were bred in the Foresterhill Animal House, University of Aberdeen.

They were fed with Thompson modified rat cube containing 14% skimmed milk (North-Eastern Agricultural Co-operative Society Ltd., Aberdeen), and water was allowed *ad libitum*. The females were caged overnight with males and the morning on which spermatozoa were found in the vaginal smears designated day 0 of pregnancy. Laparotomy was performed 24 hours before treatment to confirm pregnancy.

7-OHM-12-MBA, in crystalline form, was dissolved in olive oil (10 mg./ml.) and emulsified with saline to give a final concentration of 3 mg./ml. of 30% oil emulsion. A single intravenous injection of 2·5 mg./100 g. of maternal body weight was given into a lateral tail vein at day 8 or day 13 of pregnancy. Control groups received an equivalent volume of 30% emulsion which contained no hydrocarbon.

Metopirone, dissolved in olive oil (50 mg./ml.) was given 4 hours before treatment with 7-OHM-12-MBA, and normally in one intraperitoneal injection (40 mg.); to reduce the toxic effects two of the 8-day pregnant rats were given Metopirone in simultaneous intraperitoneal (10 mg.) and subcutaneous (30 mg.) injections before the 7-OHM-12-MBA. SKF 525-A, dissolved in sterile saline (2 mg./ml.), was injected intraperitoneally (2 mg./100 g. maternal body weight) at day 8 or day 13, 30 minutes before 7-OHM-12-MBA. The control groups received an equivalent volume of olive oil or saline and 7-OHM-12-MBA or Metopirone or SKF 525-A (see tables).

The mothers were killed with chloroform on day 20 of pregnancy. The number of dead or resorbing foetuses was noted and the survivors measured and weighed. All surviving foetuses were necropsied and at least two from each litter fixed in 95% ethanol and the skeleton stained with alizarin red. The placentae were weighed and the maternal adrenal glands fixed in 4% neutral buffered formaldehyde. Paraffin sections were stained with haematoxylin and eosin.

### RESULTS

### *Day 8 treatment*

The resorption rate in the groups treated with 7-OHM-12-MBA alone or pretreated with olive oil or saline ranged from 88% to 100% (Table I). Pretreatment with Metopirone or SKF 525-A before 7-OHM-12-MBA significantly reduced the resorption rate to 34% and 2% respectively (in each case $P < 0{\cdot}001$). In the control groups that did not receive 7-OHM-12-MBA the resorption rate was within normal limits (range 3% to 16%).

TABLE I.—*Protection by Metopirone or SKF 525-A from Embryotoxic Effects after Treatment with 7-OHM-12-MBA on Day 8 of Pregnancy*

| Pretreatment | Treatment | No. rats treated | Total No. implantations | No. resorptions (%) | No. surviving foetuses with malformations |
|---|---|---|---|---|---|
| — | 7-OHM-12-MBA | 4 | 49 | 43 (88)* | 4 |
| — | Control emulsion | 3 | 38 | 6 (16)* | 0 |
| Metopirone | 7-OHM-12-MBA | 4 | 50 | 17 (34)† | 5 |
| Olive oil | 7-OHM-12-MBA | 3 | 34 | 34 (100)† | 0 |
| SKF 525-A | 7-OHM-12-MBA | 3 | 41 | 1 (2)‡ | 0 |
| Saline | 7-OHM-12-MBA | 3 | 36 | 33 (92)‡ | 3 |
| Metopirone | — | 3 | 46 | 3 (7) | 0 |
| SKF 525-A | — | 3 | 34 | 1 (3) | 0 |

*$P < 0{\cdot}001$. †$P < 0{\cdot}001$. ‡$P < 0{\cdot}001$.

The three surviving foetuses from the group pretreated with saline, and some of those from the group pretreated with Metopirone (5/33) or treated only with 7-OHM-12-MBA (4/6), showed teratogenic effects. The malformations included exencephaly, encephalocele, spina bifida, microphthalmia, cleft palate, facial cleft, renal agenesis, eventration of abdominal viscera, talipes and kinked tail. All the surviving foetuses of the group pretreated with SKF 525-A and all those in the control groups that did not have 7-OHM-12-MBA were normal.

*Day* 13 *treatment*

With no pretreatment 7-OHM-12-MBA produced marked stunting (see Table III), and a posterior encephalocele and a spina bifida extending to the thoracic or lumbar level in 100% of surviving foetuses (Fig. 1, Table II). The alizarin red preparations revealed incomplete development of the occipital and interparietal bones and stunting and eversion of the vertebral arches; the ribs and the supra-spinous portions of the scapulae were incompletely developed and in some cases totally absent. All animals had a fairly severe cervico-thoracic lordosis.

TABLE II.—*Protection by Metopirone or SKF* 525-*A from the Teratogenic Effects after Treatment with* 7-*OHM*-12-*MBA on Day* 13 *of Pregnancy*

| | | | Number of "surviving" foetuses with | |
|---|---|---|---|---|
| Pretreatment | Treatment | No. rats treated | no visible abnormality | posterior encephalocele and spina bifida |
| — | 7-OHM-12-MBA | 5 | 0 | 55* |
| — | Control emulsion | 6 | 63 | 0* |
| Metopirone | 7-OHM-12-MBA | 5 | 61 | 0† |
| Olive oil | 7-OHM-12-MBA | 5 | 0 | 53† |
| SKF 525-A | 7-OHM-12-MBA | 6 | 57 | 0‡ |
| Saline | 7-OHM-12-MBA | 4 | 0 | 42‡ |
| Metopirone | — | 5 | 61 | 0 |
| SKF 525-A | — | 5 | 55 | 0 |

* $P < 0.001$. † $P < 0.001$. ‡ $P < 0.001$.

Pretreatment with Metopirone or SKF 525-A was highly protective (in each case $P < 0.001$) and none of the surviving foetuses showed any macroscopic abnormalities. The alizarin red preparations, however, revealed that in many of these foetuses skeletal development was incomplete: in most cases the osseous defects were minimal and chiefly involved the interparietal and occipital bones of the skull and the lower pairs of ribs. SKF 525-A was again more effective in its protective action than Metopirone: less than half of the foetuses pretreated with SKF 525-A showed minor skeletal defects whereas nearly two-thirds of the Metopirone-pretreated group were affected. The resorption rate was within normal limits (range 2% to 10%) in all groups and teratogenic effects were not produced by Metopirone or SKF 525-A alone.

*Foetal and placental weights*

The litters treated on day 13 of pregnancy with 7-OHM-12-MBA showed significant reductions in foetal and placental weights on day 20 (Table III). Metopirone and SKF 525-A pretreatment significantly increased the foetal and placental weights; the increase in placental weight was proportionately greater than the corresponding increase in foetal weight.

1

FIG. 1.—Foetus from 7-OHM-12-MBA-treated litter (left) showing marked stunting, a posterior encephalocele and a spina bifida extending to the lumbar region. Control foetus on right. × approx. 2·5.

TABLE III.—*Effects of Metopirone or SKF 525-A Pretreatment on the Foetal and Placental Weights at Day 20 after Treatment with 7-OHM-12-MBA on Day 13 of Pregnancy*

| Pretreatment | Treatment | No. of litters | Mean foetal weight g. ± S.E. (No. weighed) | Mean placental weight mg. ± S.E. (No. weighed) |
|---|---|---|---|---|
| — | 7-OHM-12-MBA | 5 | 2·60±0·13* (45) | 328±12† (55) |
| — | Control emulsion | 6 | 3·64±0·16* (50) | 497±22† (63) |
| Metopirone | 7-OHM-12-MBA | 5 | 3·02±0·18‡ (51) | 458±35§ (61) |
| Olive oil | 7-OHM-12-MBA | 5 | 2·67±0·14‡ (44) | 350±25§ (53) |
| SKF 525-A | 7-OHM-12-MBA | 6 | 3·08±0·26‖ (48) | 470±61¶ (57) |
| Saline | 7-OHM-12-MBA | 4 | 2·96±0·12‖ (32) | 323±18¶ (42) |
| Metopirone | — | 5 | 3·68±0·12 (51) | 531±25 (61) |
| SKF 525-A | — | 5 | 3·58±0·15 (45) | 523±20 (55) |

*$P < 0{\cdot}001$. †$P < 0{\cdot}001$. ‡$P < 0{\cdot}02$.

§$P < 0{\cdot}001$. ‖$P \approx 0{\cdot}1$. ¶$P < 0{\cdot}001$.

*Maternal adrenal glands*

The maternal adrenal glands were assessed independently by three separate observers. SKF 525-A pretreatment completely protected the adrenal from 7-OHM-12-MBA. With Metopirone, protection was less complete and nearly one-third of the rats pretreated at day 8 or day 13 showed single scattered necrotic cells or small foci of necrosis in the inner zones of the cortex. A close correlation was found between the degree of protection of the maternal adrenal glands and the protection of the foetuses.

DISCUSSION

Metopirone and SKF 525-A protect the Sprague-Dawley rat from the embryotoxic and teratogenic effects of 7-OHM-12-MBA. Metopirone is less effective than SKF 525-A, but the dose of SKF 525-A was related to the weight of the mothers whereas a constant dose of Metopirone was given. The resorption rate in two of the unprotected groups treated with 7-OHM-12-MBA at day 8 was somewhat less (88% and 92%) than that expected from our previous findings (Currie *et al.*, 1970). Some or all of the surviving foetuses in these groups, and in the Metopirone-pretreated group, were malformed. The types of malformation were similar to those found at day 8 with lower doses of 7-OHM-12-MBA (Currie *et al.*, 1970), and it seems probable that some or all of the foetuses which resorb before term are malformed.

The proportionately greater increase in the placental weight as compared with foetal weight in the groups pretreated with Metopirone and SKF 525-A at day 13 of pregnancy is of interest. Robson and Sullivan (1966) have shown that disturbances of placental function may play an important part in the teratogenic actions of some compounds, and it is possible that 7-OHM-12-MBA may have significant effects on placental development and function at day 13 of pregnancy.

Metopirone and SKF 525-A also protect against the adrenocorticolytic actions of 7-OHM-12-MBA in the mature rat, and in our experiments there was a close correlation between the degree of protection of foetuses and of the maternal adrenal glands. It is also of interest that SKF 525-A protects the foetal adrenal gland (Bird, Crawford and Currie, 1970). Prevention of the adrenocorticolytic effects with these compounds is currently assumed by some workers to depend only on their interference with the hepatic metabolism of 7-OHM-12-MBA. Metopirone, an inhibitor of adrenal 11-$\beta$ hydroxylation (Chart *et al.*, 1958), is thought to stimulate the drug-metabolizing enzyme systems of the liver microsomes thus enhancing the inactivation of 7-OHM-12-MBA (Boyland and Sims, 1967; Wheatley, 1968*a*). However, Jellinck and Garrett (1969), while confirming the *in vivo* effectiveness of Metopirone in preventing DMBA-induced adrenal necrosis, were unable to show any appreciable difference in the metabolism of DMBA by liver extracts prepared from Metopirone-pretreated rats. By contrast SKF 525-A is a potent inhibitor of the hepatic microsomal enzymes (Axelrod, Leichtenthal and Brodie, 1954; Cooper, Axelrod and Brodie, 1954), and Huffman and Azarnoff (1967) have shown that it also inhibits corticosteroid synthesis by the rat adrenal gland. It is possible that the ultimate active adrenocorticolytic agent may be formed within adrenocortical cells. Whatever the case it is virtually certain that a metabolite of 7-OHM-12-MBA is the ultimate adrenocorticolytic agent (Wheatley and Sims, 1969), and the results here reported indicate that the ultimate embryopathic agent is also a metabolite of 7-OHM-12-MBA and not 7-OHM-12-MBA itself. It may be that the ultimate embryopathic agent is formed within the affected foetal tissues, and it remains to be shown whether it and the ultimate adrenocorticolytic agent are the same compound.

This investigation was supported by a grant to A.R.C. from the Scottish Hospital Endowments Research Trust. The 7-OHM-12-MBA was kindly supplied by Dr. Peter Sims, Chester Beatty Research Institute, Royal Cancer Hospital, London. Our thanks are also due to Mrs. Marget Inglis, Miss Barbara Cruden and Mr. G. Milne for technical assistance.

### REFERENCES

Axelrod, J., Leichtenthal, J. and Brodie, B. B.—(1954) *J. Pharmac. exp. Ther.*, **112**, 49.

Bird, C. C., Crawford, A. M. and Currie, A. R.—(1970) *Nature, Lond.*, in press.

Boyland, E. and Sims, P.—(1967) *Biochem. J.*, **104**, 394.

Boyland, E., Sims, P. and Huggins, C.—(1965) *Nature, Lond.*, **207**, 816.

Chart, J. J., Sheppard, H., Allen, M. J., Bencze, W. L. and Gaunt, R.—(1958) *Experientia*, **14**, 151.

Cooper, J. R., Axelrod, J. and Brodie, B. B.—(1954) *J. Pharmac. exp. Ther.*, **112**, 55.

Currie, A. R., Bird, C. C., Crawford, A. M. and Sims, P.—(1970) *Nature, Lond.*, **226**, 911.

Currie, A. R., Helfenstein, J. E. and Young, S.—(1962) *Lancet*, ii, 1199.

Dao, T. L. and Tanaka, Y.—(1963) *Proc. Soc. exp. Biol. Med.*, **113**, 1199.

Jellinck, P. H. and Garrett, T.—(1969) *Experientia*, **25**, 799.

Huffman, D. H. and Azarnoff, D. L.—(1967) *Steroids*, **9**, 41.

Robson, J. M. and Sullivan, F. M.—(1966) *J. Physiol., Lond.*, **194**, 717.

Wheatley, D. N.—(1968*a*) *Endocrinology*, **82**, 1217.—(1968*b*) *Br. J. exp. Path.*, **49**, 44.

WHEATLEY, D. N., HAMILTON, A. G., CURRIE, A. R., BOYLAND, E. AND SIMS, P.—(1966) *Nature, Lond.*, **211**, 1311.
WHEATLEY, D. N. AND SIMS, P.—(1969) *Biochem. Pharmac.*, **18**, 2583.
WONG, T. W. AND WARNER, N. E.—(1964) *Endocrinology*, **74**, 284.


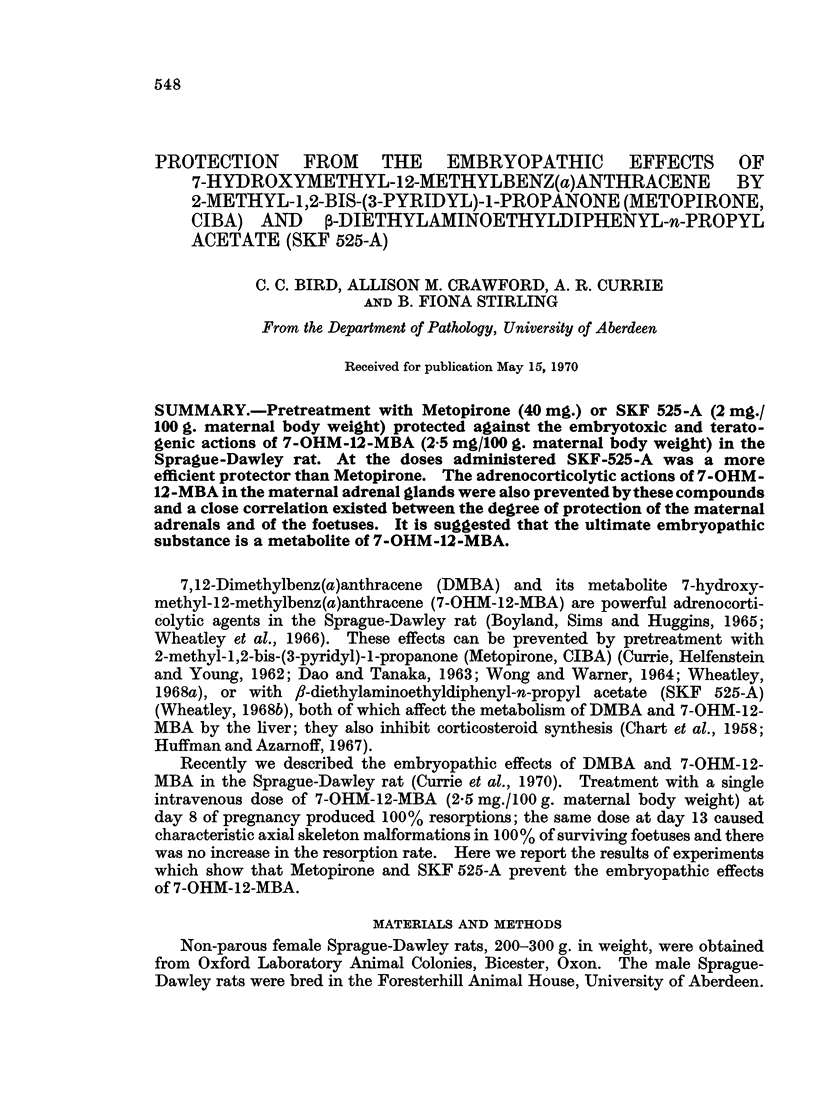

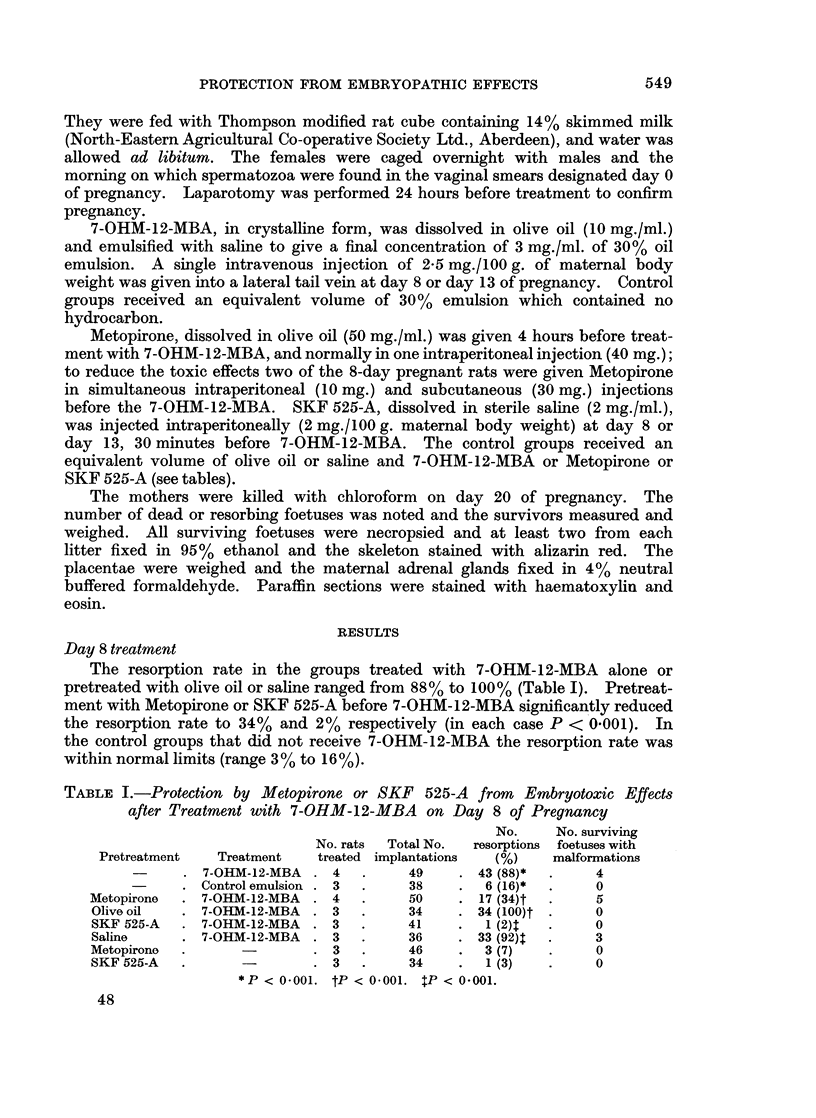

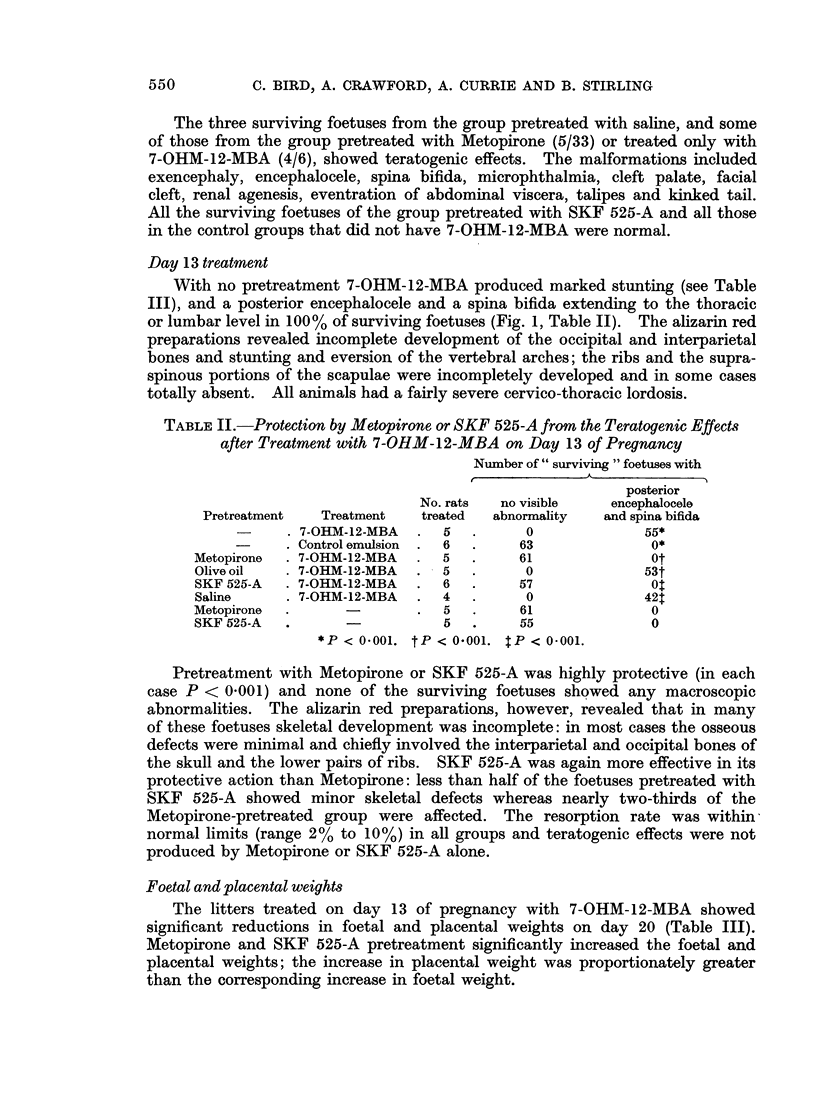

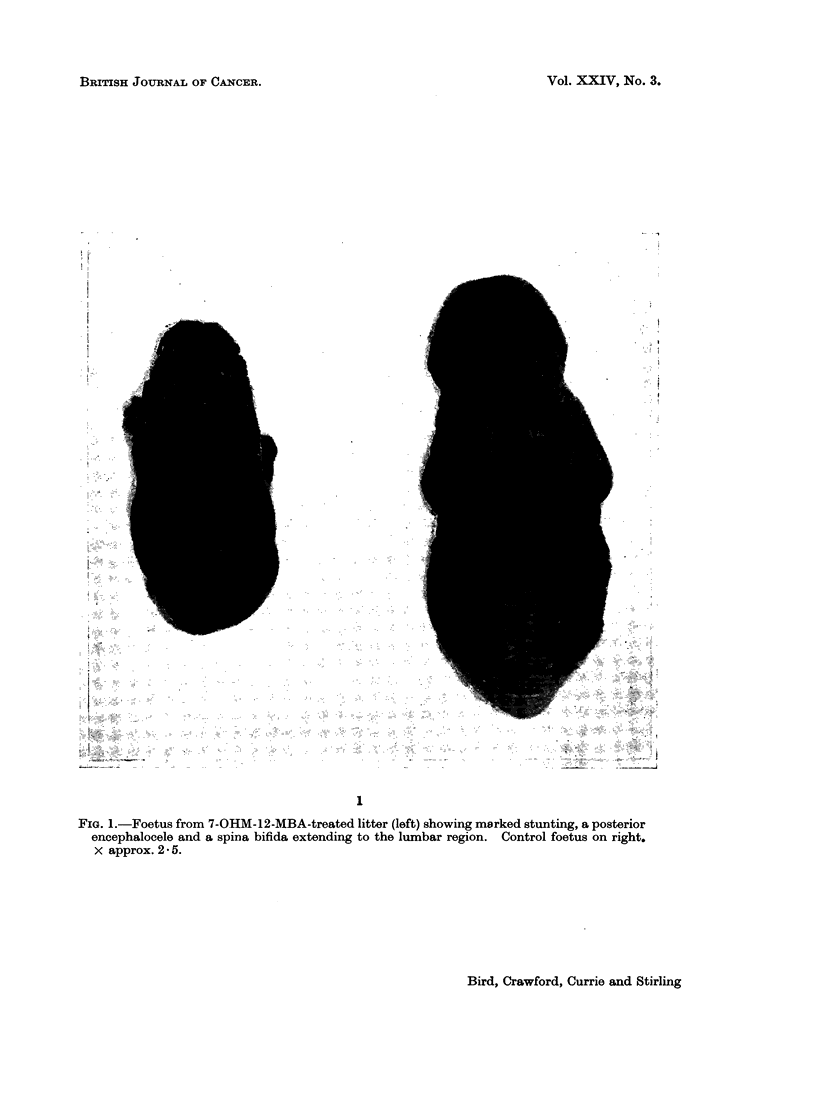

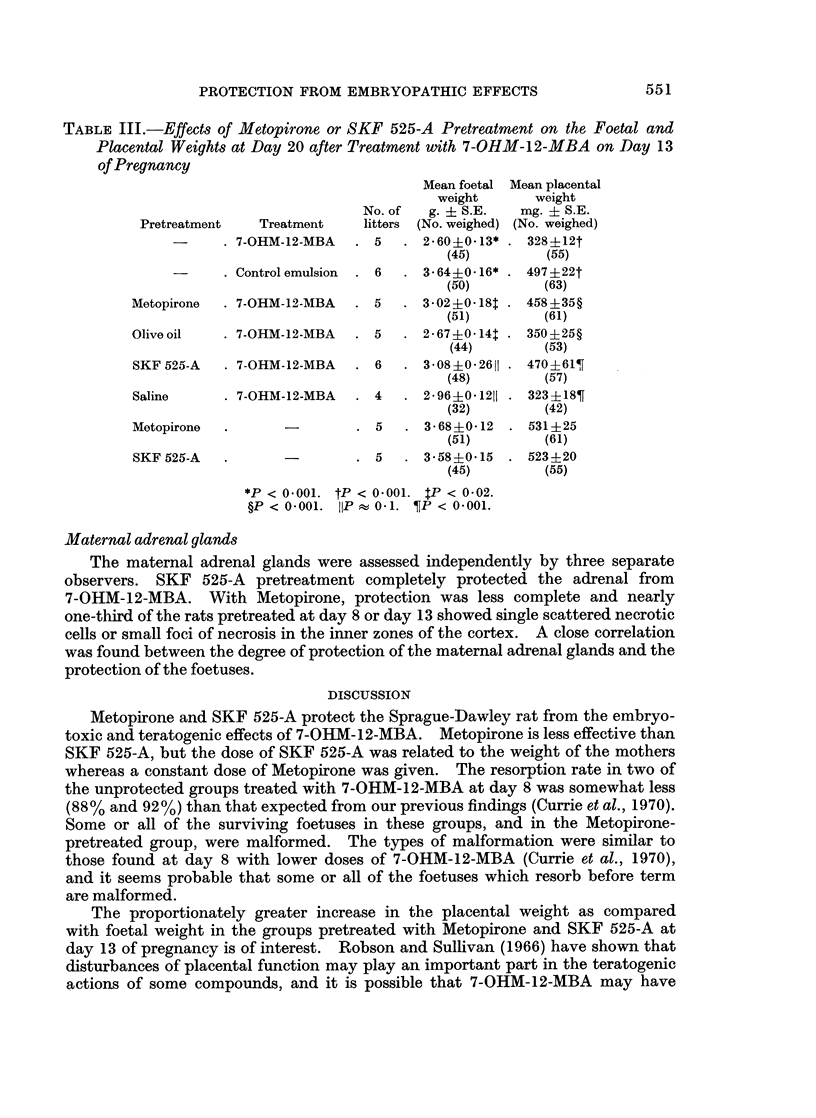

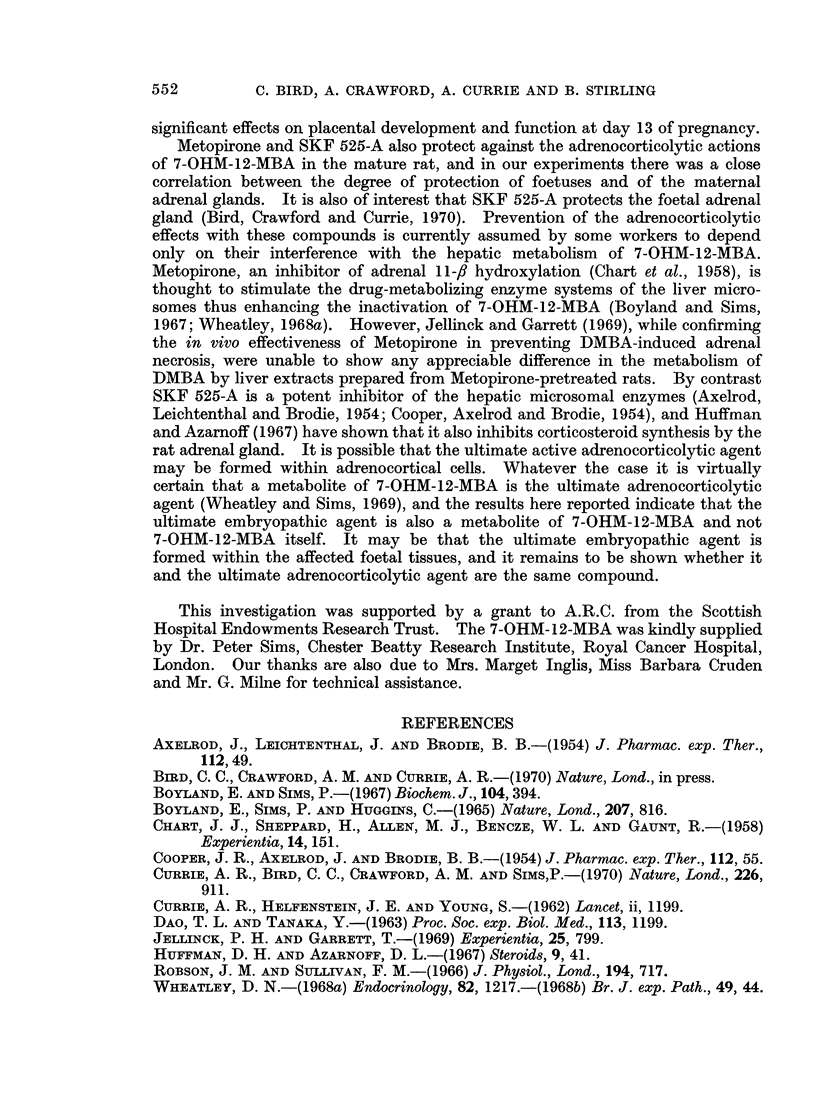

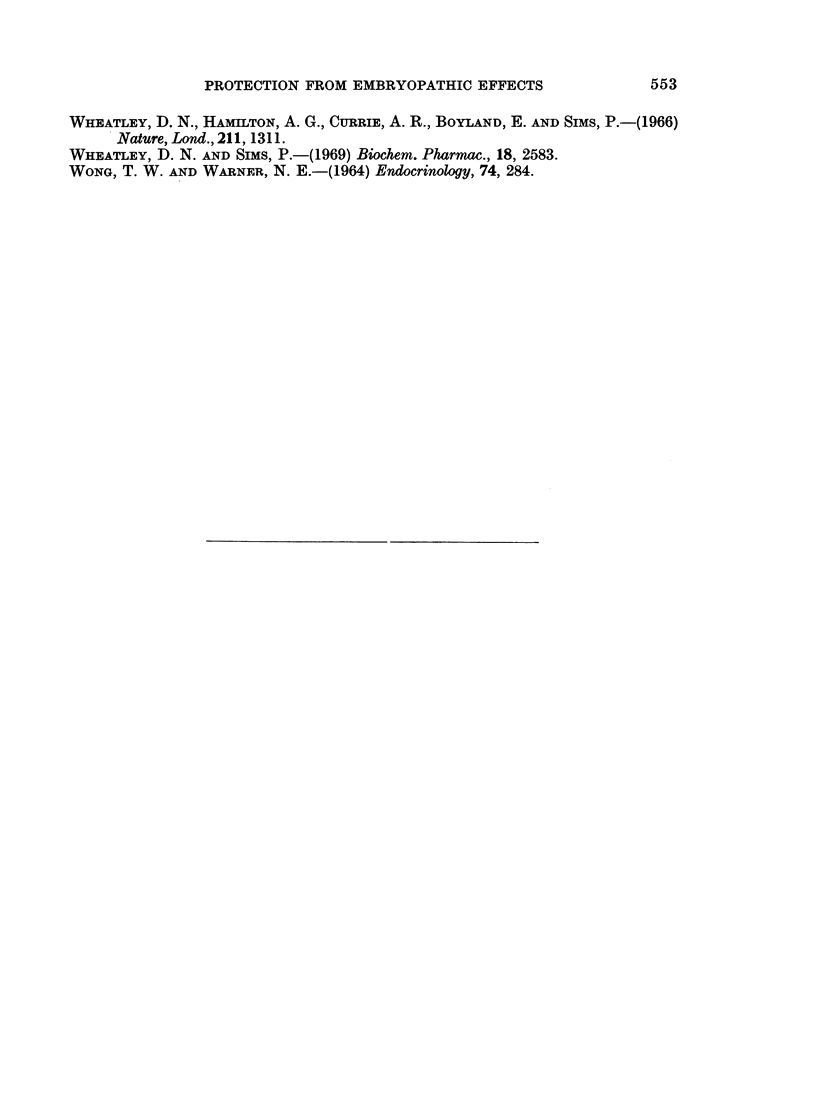


## References

[OCR_00378] Boyland E., Sims P., Huggins C. (1965). Induction of adrenal damage and cancer with metabolites of 7,12-dimethylbenz(a)anthracene.. Nature.

[OCR_00380] CHART J. J., SHEPPARD H., ALLEN M. J., BENCZE W. L., GAUNT R. (1958). New amphenone analogs as adrenocortical inhibitors.. Experientia.

[OCR_00390] CURRIE A. R., HELFENSTEIN J. E., YOUNG S. (1962). Massive adrenal necrosis in rats caused by 9,10-dimethyl-1,2-benzanthracene and its inhibition by metyrapone.. Lancet.

[OCR_00391] Jellinck P. H., Garrett T. (1969). Metabolism of metopirone and 3-(1,2,3,4-tetrahydro-1-oxo-2 naphthyl)-pyridine in relation to DMBA induced adrenal necrosis.. Experientia.

[OCR_00405] WONG T. W., WARNER N. E. (1964). INHIBITION OF DIMETHYLBENZANTHRACENE-INDUCED ADRENAL CORTICAL NECROSIS.. Endocrinology.

[OCR_00396] Wheatley D. N. (1968). Action of drugs affecting the functioning of the adrenal cortex on adrenal necrosis induced by 7,12-dimethylbenz(alpha)anthracene and its 7-hydroxymethyl derivative in rats.. Endocrinology.

[OCR_00404] Wheatley D. N., Sims P. (1969). Comparison of the efficacy of pretreatment protection against adrenal necrosis induced by 7-hydroxymethyl-12-methylbenz(a)anthracene and by 7-methyl-12-methylbenz(a)anthracene in rats.. Biochem Pharmacol.

